# Soil-Transmitted Helminth Infections and Nutritional Status in School-age Children from Rural Communities in Honduras

**DOI:** 10.1371/journal.pntd.0002378

**Published:** 2013-08-08

**Authors:** Ana Lourdes Sanchez, Jose Antonio Gabrie, Mary-Theresa Usuanlele, Maria Mercedes Rueda, Maritza Canales, Theresa W. Gyorkos

**Affiliations:** 1 Department of Community Health Sciences, Brock University, St. Catharines, Ontario, Canada; 2 School of Microbiology, National Autonomous University of Honduras, Tegucigalpa, Honduras; 3 Division of Clinical Epidemiology, Research Institute of the McGill University Health Centre, and Department of Epidemiology, Biostatistics and Occupational Health, McGill University, Montreal, Quebec, Canada; University of Oklahoma Health Sciences Center, United States of America

## Abstract

**Background:**

Soil-transmitted helminth (STH) infections are endemic in Honduras and efforts are underway to decrease their transmission. However, current evidence is lacking in regards to their prevalence, intensity and their impact on children's health.

**Objectives:**

To evaluate the prevalence and intensity of STH infections and their association with nutritional status in a sample of Honduran children.

**Methodology:**

A cross-sectional study was done among school-age children residing in rural communities in Honduras, in 2011. Demographic data was obtained, hemoglobin and protein concentrations were determined in blood samples and STH infections investigated in single-stool samples by Kato-Katz. Anthropometric measurements were taken to calculate height-for-age (HAZ), BMI-for-age (BAZ) and weight-for-age (WAZ) to determine stunting, thinness and underweight, respectively.

**Results:**

Among 320 children studied (48% girls, aged 7–14 years, mean 9.76±1.4) an overall STH prevalence of 72.5% was found. Children >10 years of age were generally more infected than 7–10 year-olds (*p* = 0.015). Prevalence was 30%, 67% and 16% for *Ascaris*, *Trichuris* and hookworms, respectively. Moderate-to-heavy infections as well as polyparasitism were common among the infected children (36% and 44%, respectively). Polyparasitism was four times more likely to occur in children attending schools with absent or annual deworming schedules than in pupils attending schools deworming twice a year (*p*<0.001). Stunting was observed in 5.6% of children and it was associated with increasing age. Also, 2.2% of studied children were thin, 1.3% underweight and 2.2% had anemia. Moderate-to-heavy infections and polyparasitism were significantly associated with decreased values in WAZ and marginally associated with decreased values in HAZ.

**Conclusions:**

STH infections remain a public health concern in Honduras and despite current efforts were highly prevalent in the studied community. The role of multiparasite STH infections in undermining children's nutritional status warrants more research.

## Author Summary

Soil-transmitted helminth (STH) infections are endemic in Honduras but their impact on children's health is not well studied. With the purpose of determining the prevalence and intensity of STH infections and their association with nutritional status in a sample of Honduran children, a cross-sectional study was undertaken in 2011. School-age children were enrolled, and in addition to demographic data, blood and stool samples and anthropometric measurements were obtained to determine nutritional status and STH infection. The overall STH prevalence among 320 studied children was 72.5% and almost half of the infected children harboured multiple parasites. Polyparasitism was more likely to occur in children attending schools with absent or annual deworming schedules than in pupils attending schools deworming twice a year. Prevalence by species was 30%, 67% and 16% for *Ascaris*, *Trichuris* and hookworms, respectively. Infections of moderate to heavy intensity as well as multiparasite infections were significant predictors of decreased weight-for-age scores in children ages 7–10 years after controlling for key confounders. Sustainable efforts to control STH infections in Honduras are required. Future research providing more insight on the nutritional impact of polyparasitic STH infections in childhood is necessary.

## Introduction

Honduras is among 30 countries in the Americas that are endemic for soil-transmitted helminth (STH) infections, which are caused by four species of intestinal nematodes: the common roundworm, *Ascaris lumbricoides*; the whipworm, *Trichuris trichiura*; and the hookworms, *Necator americanus* and *Ancylostoma duodenale* [1]. The health impact of these infections is more dramatic in children, for whom STH show a particular predilection [2] partly due to their differential exposure to contaminated soil. Health adverse effects such as anemia, growth stunting, protein-calorie malnutrition, fatigue, and poor cognitive development tend to occur and persist in populations affected by STH [3], and all too often, helminth infections are seen as normal and unavoidable part of life in endemic populations [4].

According to the World Health Organization (WHO), two thirds of Honduran children aged 1–14 years require preventive chemotherapy (PC) for STH [1]. In fact, the Preventive Chemotherapy and Transmission Control (PCT) databank of the WHO estimates that 2.6 million Honduran children (769,405 pre-school and 1,832,476 school-age children) are at risk for STH transmission therefore requiring regular administration of anthelminthic drugs [5]. Organized STH control activities in the country began in 1998 with the establishment of the Healthy Schools Program, a collaborative effort between the ministry of health, the ministry of education and the World Food Program [6]. By 2001, Honduras had started subnational control activities [1], [7] and these soon evolved into a national program guided by the recommendations outlined in the World Health Assembly resolution 54.19 [6], [8]. Although the goal of providing anthelminthic medication in a regular manner to at least 75% of all school-age children at risk has yet to be attained [1], [5], [6], [7], Honduras' efforts of tackling STH transmission are, nevertheless, commendable. An important complement to these efforts would be to undertake a complete situation analysis at subnational levels; one that would establish baseline data in terms of prevalence as well as transmission risk factors so priority areas can be identified and intervention efforts tailored to specific populations [9]. Additionally, to effectively monitor the success of PC and other interventions, studies assessing STH-associated morbidity in infected children are needed in Honduras.

Based on scientific evidence linking STH morbidity with worm burden (*i.e.*, the number of adult parasites inhabiting the intestine [10]), the elimination of moderate and heavy infections is the target of PC programs [1]. In addition to worm burden, polyparasitism -the concurrent infection with multiple parasite species- has also been associated with children's malnutrition [11], [12]. As well, even when recent data is scarce, some studies have reported that even light infections may impose a threat to children's health [13], especially if living in endemic communities with poor nutritional status [10], [11], [14]. Hence, the overlap of poverty, malnutrition and STH endemicity in some populations may obscure the true effect of these helminthiases in childhood health and accordingly, more research is needed to fully appreciate the burden of these infections on people living in these areas [15].

With this in mind, the aim of this study was to investigate the prevalence and intensity of STH infections in a sample of Honduran school-aged children and examine whether STH are negatively associated with malnutrition and anemia.

## Methods

### Ethics statement

The present study was nested within a parent study entitled ‘Gender and parasitic diseases: Integrating gender analysis in epidemiological research on parasitic diseases to optimize the impact of prevention and control measures’ (principal investigator, T. W. Gyorkos, McGill University, Canada) and both received ethics clearance from McGill University Health Centre, Montreal, QC (file number MUHC 10 -175 – PED Nov. 23rd 2010) and Brock University, St. Catharines, ON (file number - BU 10–161 – Sanchez/Gyorkos Jan 13th 2011). In the absence of an institutional ethics board in the participating academic unit of the Honduran university, the Ethics Officer of the Masters Program in Infectious and Zoonotic Diseases (MEIZ) of the School of Microbiology, National Autonomous University of Honduras, reviewed the protocol and provided clearance (file number OF-MEIZ- 001-2011).

As the study population comprised minors, both parental consent and children's assent were required prior to enrolment of children. Parents and guardians of children in grades 3–5 were invited to an information session in which the study's objectives, benefits and risks were fully explained. Parents and guardians who gave oral consent were presented with an information package containing a detailed lay description of the study, an invitation to participate and a consent form for their signature. All parents or guardians consenting for their children to participate signed the informed consent form. Children whose parents consented were invited to participate in the study during sessions held at the schools and those who expressed assent in responding to a questionnaire, providing a stool and blood sample and allowing the collection of anthropometric measurements were then enrolled in the study. Children assents were obtained verbally and documented through a child assent form. Also, since the study was undertaken during class time at participating schools, authorizations from schools' Principals were sought in advance and only schools with such authorizations were approached for enrolment. Laboratory reports were issued with accompanying lay interpretations and recommendations. Also, parents of children with STH infections were offered anti-parasitic treatment for their child. If agreed, albendazole tablets (400 mg) were administered to the child by the school teacher or parent. A “deworming tracking card” was issued for each child. Parents and teachers were encouraged to keep track of the children's deworming treatment in order to either avoid missing the school's annual or bi-annual treatment or prevent excessive treatments in case deworming was offered by third parties (*e.g.*, international or national medical brigades, faith-based missions, etc.).

### Study design and determination of sample size

Both the parent study and present study were school-based, cross-sectional studies, designed as explorative and hypothesis generating studies.

For the parent study, power and sample size determination were performed utilizing the PS software (version 3.0, January 2009, by William D. DuPont and Walton D. Plummer Jr.). This was based on a two-sided chi-square test to compare STH infection between boys and girls. Using previous studies in Peru as a reference [16] it was assumed that half of the children in this school-age group will be male and that the prevalence of any STH would be 50% in males (a conservative estimate). An estimated design effect of 2.7 was used with a significance level of 0.05. A total of 314 participants were therefore needed to detect a minimum risk ratio of 1.5 with 80% power. The present study was bound by this sample size determination.

### Study area and population

This study was implemented during February and March 2011 with the collaboration of the National University of Agriculture (UNA) located in the city Catacamas, in the municipality of the same name (14°51′35.46″; N 85°53′58.19″W) in the Department of Olancho, about 210 km north-east of the capital of Honduras, Tegucigalpa. Geographically, Catacamas is the largest municipality in the country and is nested in a fertile valley at approximately 450 m above sea level. Catacamas municipality consists of the urban core (Catacamas proper) and 14 main villages which in turn are comprised of smaller 339 hamlets. Catacamas human development index (HDI) value for 2009 was 0.675 [17], slightly over to that of Honduras (0.625 for 2011) [18]. However, 60% of Catacamas' population resides in rural areas, the majority lacking public services such as electricity, potable water and indoor plumbing. As means of livelihood inhabitants engage in mixed agricultural farming, rearing animals such as cattle, pigs and poultry and growing crops such as corn, beans, coffee and vegetables. Others work as traders or labourers while a few work in public or private service [19].

The following nine surrounding rural communities (most between 2–3 hour driving distance from the city) were visited as potential study-sites: Colonia de Poncaya, Las Lomas de Poncaya, Las Parcelas, Corosito de Poncaya, El Cerro del Vigía, El Hormiguero, Santa Clara, Los Lirios and Campamento Viejo. The combined eligible school population was 445 children. Schools located in those communities were identified and principals contacted by UNA's personnel to inform them about the study and obtain authorization to approach the school and potentially enrol their pupils. As well, information was obtained in regards to school enrolment and status of their deworming program, if any. Schools which had provided deworming treatment within the last three months were not eligible for the study.

### Study sample

The target participants for the study were children in grades 3–5 (aged 9–11) since STH infections, especially *A. lumbricoides* and *T. trichiura* tend to peak at this age [1]. Also, at this age children are old enough to understand survey questions and provide basic information.

### Data collection

Using a pre-tested, 30-minute, face-to-face standardized questionnaire in Spanish, the Gender Study collected demographic and epidemiological data as well as children's living conditions and knowledge regarding STH infections. From these data, the present study extracted children's general demographics (name, date of birth, age, and sex of the child), STH and deworming history, self-reported living conditions (household's type of floor, water access and type and use of sanitary facilities), and the possession of major home appliances.

### Assessment of nutritional status

Body weight and height measurements were taken for each child to calculate anthropometric indicators. Weights were taken using a digital electronic balance to the nearest 0.1 kg while children were wearing school uniforms and without shoes. Height was taken to the nearest 0.1 cm using a height pole mounted on the wall. In order to minimize intra-individual errors, all measurements were taken twice by different researchers and the average value calculated and used thereof.

Age, height and weight were then used to calculate the following indicators: a) height-for-age Z-score (HAZ) to assess stunting; b) weight-for-age Z-score (WAZ) to assess underweight; and c) body-mass-index-for-age Z-score (BAZ) to assess thinness. Calculations were done with the WHO AnthroPlus software version 1.04 (WHO, Geneva, Switzerland) using the WHO international reference values (available at http://www.who.int/growthref/tools/en/). Because of its inability to differentiate between relative height and body mass, WAZ is not recommended for the assessment of growth beyond childhood (>10 years of age) [19]. Therefore, BAZ was used as a complement to HAZ. These indicators are recommended by the WHO as they provide an assessment of the child's nutritional status in comparison with a healthy reference population [20], [21]. According to the 2007 WHO growth reference for school-aged children and adolescents, stunting, underweight and thinness are defined as <−2 standard deviations (SD) HAZ, WAZ and BAZ, respectively [20].

### Stool collection and parasite determination

A single fecal sample was collected from each child and samples were taken to UNA's laboratory for analysis on the same day using the Kato-Katz technique [22] with a template of 41.7 mg, as recommended by the WHO [23]. Kato-Katz templates were obtained from Vestergaard-Frandsen Disease Control Textiles (Vestergaard Frandsen SA, Aarhus, Denmark). Kato–Katz slides were examined microscopically in a systematic manner within 30–60 min of preparation; helminth eggs counted for each parasite species and the number thus obtained multiplied by a factor of 24 in order to get the number of eggs per gram of feces (epg). Egg counts were utilized to classify infection intensities into light, moderate, or heavy infections as follows, respectively: for *A. lumbricoides*, 1–4,999 epg, 5,000 – 49,999 epg and ≥50,000 epg; for *T. trichiura*, 1–999 epg, 1,000–9,999 epg and ≥10,000 epg; and for hookworms, 1–1,999 epg, 2,000–3,999 epg and ≥4,000 epg [1], [24].

### Haematological and protein analysis

Haematological analyses were done using the BC – 3000Plus AutoHematology Analyzer (Mindray Medical Instrumentation, USA) in a private medical laboratory contracted in Catacamas. Anemia was determined when children aged 6–11 years had hemoglobin (Hb) values <11.5 g/dL or hematocrit (Hct) <34%. For children aged 12–14 years, these values were Hb <12 g/dL or Hct <36% [25]. Total serum protein concentrations were measured by the Biuret method and children were considered within the reference values if concentrations were within 6–8.5 g/dL [26].

### Statistical analyses

Data were entered by a researcher into Microsoft office Excel spreadsheet 2007 (Microsoft) and verified for accuracy (compared with data in questionnaires) by a different researcher. Data were cleaned by checking for errors and missing values. Statistical analyses were done using IBM, SPSS Statistics ver. 20 (IBM. Somers, NY). Descriptive statistics for continuous variables and frequency (proportions) for categorical variables were used to describe the characteristics of the study population. Weight and height measurements were subjected to a reliability test and the inter observer technical error of measurements was assessed using the Mueller and Martorell method [27]. Differences in proportions for categorical variables (*e.g.*, age group, sex of the child, stunting, thinness, underweight and anemia) were calculated using Chi square test of independence. Differences in mean values for continuous variables (*e.g.*, HAZ, WAZ, BAZ, total proteins, Hb and Hct) were assessed using the student *t*-test analysis.

Since STH clinical importance is generally associated with increased worm burden, infections of moderate and heavy intensity were merged into one category “moderate-to-heavy”. This was also useful for computational reasons since those infections were in minority among studied children. Also, to assess polyparasitism, a category termed “infection status” was created to denote conditions of non-infected, monoparasitism or polyparasitism (co-infections with 2 or 3 STH). One-way ANOVA was used to analyze differences in anthropometric mean Z-scores of the study population by infection status and by infection intensity (negative, light and moderate-to-heavy) of each parasite species.

A generalized estimating equations (GEE) approach was used to construct both multivariable linear and logistic regression models to account for possible within-school data correlation (clustering at the school level). For these models, intensity of infection was not analyzed by parasite species. Rather, infection categories “negative-to-light” and “moderate-to-heavy” were created to denote individuals with such infections by any of the three parasites under study. Linear regression models to test for associations between anthropometric indicators and intensity of infection categories were constructed adjusting for age, sex, socio-economic status (SES) and anemia. Similar models were done to test for association between those indicators and infection status. Using principal component analysis (PCA), the SES variable was constructed from five factors: type of floor, access to tap water, having a toilet, having a television set and having a fridge. Separate logistic regression models were constructed to assess associations between stunting and thinness odds and STH intensity of infection and infection status adjusting for age stratum (7–10 or >10 years of age), sex and SES. Odds ratios (OR) were determined with 95% confidence intervals (CI = 95%).

## Results

### Study participation and characteristics of the study population

Of the nine visited, seven schools were enrolled in the study: Colonia de Poncaya, Las Lomas de Poncaya, Las Parcelas, Corosito de Poncaya, El Cerro del Vigía, El Hormiguero, and Campamento Viejo. The reasons for not including the two remaining were: recent deworming treatment (Santa Clara n = 26) and time-constraints to complete questionnaires and measurements (Los Lirios n = 19). (Los Lirios' children, however, were examined for STH and treatment provided if needed). Thus, the number of eligible participants in grades 3 to 5 among participating schools was 400 children. The parents of 368 (92%) children provided written informed consent for their children to participate and almost all (357, 97%) children assented to be enrolled. After enrolment, 37 participants were dropped from the study due to insufficient or no stool sample (n = 20), or unreliable Kato-Katz results that could not be repeated (n = 17). Also, five children declined blood collection but they were kept in the study since their remaining data was complete. The final study sample was 320 children aged 7–14 years (mean 9.76±1.4) and 154 (48%) were girls. Demographic, household and nutritional characteristics of the study sample are shown in Table 1. Additionally, habitual or occasional open defecation was reported by 15.6% and 12.8% of the children, respectively. As for STH history, 58.1% of the children reported having expelled ‘worms’ in the past and 85.9% recalled having received deworming treatment sometime in the past but not recently.

**Table 1:** Demographic, household and nutritional characteristics of the study sample according to STH infection.

				Infection status			
	All children	Positive for any STH		Non-infected	Mono-parasitism	Poly-parasitism	
	N = 320	N = 232		N = 88	N = 129	N = 103	
Characteristics	n (%)^a^	n (%)^a^	*p-value* b	n (%)^a^	n (%)^a^	n (%)^a^	*p-value* c
**Sex**							
Girls	154 (48.1)	110 (47.4)	0.708	44 (50.0)	69 (53.5)	41 (39.8)	0.107
**Age**							
7–10 years old	234 (73.1)	161 (69.4)	0.016	73 (83.0)	88 (68.2)	73 (70.9)	0.046
>10 years old	86 (26.9)	71 (30.6)		15 (17.0)	41 (31.8)	30 (29.1)	
**Household**							
Earthen floor	118 (36.9)	99 (42.7)	<0.001	19 (21.6)	50 (38.8)	49 (47.6)	0.001
Access to tap water	276 (86.3)	194 (83.6)	0.029	82 (93.2)	111 (86.0)	83 (80.6)	0.042
Having toilet	167 (52.2)	123 (53)	0.618	44 (50.0)	79 (61.2)	44 (43.1)	0.021
Owning TV set	154 (48.1)	100 (43.1)	0.003	54 (61.4)	64 (49.6)	36 (35.0)	0.001
Owning fridge	119 (37.2)	74 (31.9)	0.002	45 (51.1)	46 (35.7)	28 (27.2)	0.003
**School deworming schedule**							
None-once/year	246 (76.9)	177 (76.3)	0.767	69 (78.4)	84 (65.1)	93 (90.3)	<0.001
Twice/year	74 (23.1)	55 (23.7)		19 (21.6)	45 (34.9)	10 (9.7)	
**Nutritional indicators**							
Mean HAZ score (n = 320)	−0.44 (0.96)	−0.50 (0.97)	0.097	−0.30 (0.94)	−0.40 (0.95)	−0.60 (0.97)	0.071
Mean BAZ score (n = 320)	−0.04 (0.99)	−0.06 (1.00)	0.492	0.02 (0.98)	0.05 (1.06)	−0.17 (0.93)	0.202
Mean WAZ^d^ score (n = 234)	−0.09 (0.92)	−0.15 (0.94)	0.131	0.05 (0.89)	0.04 (0.92)	−0.33 (0.92)	0.012
Stunted (< −2 SD HAZ)	18 (5.6)	14 (6.0)	0.788	4 (4.5)	5 (4.3)	9 (7.7)	0.475
Thin (< −2 SD BAZ)	7 (2.2)	6 (2.6)	0.678	1 (1.1)	3 (2.6)	3 (2.6)	0.731
Underweight (< −2 SD WAZ)	3 (1.3)	3 (1.9)	0.554	0 (0)	0 (0)	3 (3.7)	0.057
Mean total serum proteins (g/dL)	7.48 (0.47)	7.47 (0.47)	0.482	7.51 (0.48)	7.45 (0.45)	7.49 (0.49)	0.663
Mean hemoglobin (g/dL)	12.94 (0.76)	12.95 (0.77)	0.607	12.91 (0.74)	12.99 (0.83)	12.93 (0.71)	0.735
Mean hematocrit (%)	38.99 (2.11)	39.07 (2.15)	0.255	38.77 (2.02)	39.01 (2.31)	39.13 (1.98)	0.475
Anemia	7 (2.2)	6 (1.9)	0.678	1 (1.1)	4 (3.5)	2 (1.7)	0.478

a For continuous variables, values in parentheses are the standard deviation.

b Independent *t*-test used for continuous variables and chi-square test used for categorical variables.

c One-way ANOVA used for continuous variables and chi-square test used for categorical variables.

d WAZ calculation is not recommended for children >10 years of age.

Five of the seven schools enrolled in the study had ongoing deworming programs, some starting as far back as 2007. Frequency of deworming was twice a year for two schools and once a year for three schools. The last deworming treatment had been within the last 4–6 months for four schools and two years for the remaining one. There was no statistical difference between overall infection with any STH and schools' deworming schedule (*p* = 0.767).

### Parasitic profile of the studied children

#### Prevalence of STH infections

A total of 232 of 320 children studied were infected with one or more intestinal helminths, for an overall point STH prevalence of 72.5% (95% CI = 67.6–77.4). Specifically, the prevalence for *T. trichiura*, *A. lumbricoides*, and hookworms was 66.9%, 30.3% and 15.9%, respectively. Children >10 years of age had twice the odds of being infected with any of the three STH than younger children (OR = 2.146, 95% CI = 1.2–4.0, *p* = 0.016). A statistical difference was not observed in terms of overall STH positivity and the sex of the children (*p* = 0.708). However, a closer look at infection by species revealed that boys of any age were twice as likely as girls to be infected by hookworms (OR = 2.076, 95% CI = 1.1–3.9, *p* = 0.022).

#### Intensity of STH infections

The majority of *T. trichiura* and hookworm infections were light intensity (73.4% and 94.1%, respectively) whereas for *Ascaris* the proportion of such infections was 40.2%. Overall, over one third of all infections (84 of 232, 36.2%) were moderate-to-heavy. Specifically, infections of heavy intensity accounted for 6.2%, 1.9% and 3.9% of the cases of ascariasis, trichuriasis and hookworm infection, respectively.

#### Polyparasitism

As shown in Table 2, of 232 infected children, 103 (44.4%) were polyparasitized. Of the latter, 27 (26.2%) harboured triple infections. *T. trichiura* prevailed across all combinations and it was found more frequently associated with *A. lumbricoides* than with hookworms.

**Table 2:** Proportion of cases with monoparasitism or polyparasitism among 232 infected children.

Type of infection	N° species	Species associated	Cases (%)
**Monoparasitism**	**1 species (n = 129)**	*T. trichiura*	113 (87.6)
		*A. lumbricoides*	9 (7.0)
		Hookworms	7 (5.4)
		**Total monoparasitism**	**129 (55.6)**
**Polyparasitism**	**2 species (n = 76)**	*T. trichiura* & *A. lumbricoides*	59 (77.6)
		*T. trichiura* & Hookworms	15 (19.7)
		*A. lumbricoides* & Hookworms	2 (2.6)
		**Sub-total**	**76 (73.8)**
	**3 species (n = 27)**	All three STH species	**27 (26.2)**
		**Total polyparasitism**	**103 (44.4)**

A statistical difference between schools' deworming schedule and polyparasitism was observed (Table 1). Children attending schools with absent or once-a-year deworming schedule were almost four times as likely to be polyparasitized (OR = 3.85, 95% CI = 1.97–7.50, *p*<0.001). Indeed, only 10% of children attending schools providing deworming twice a year harboured multiparasite infections.

### Children's nutritional status

Replicate weight and height measurements showed high reliability when tested for the inter-observer technical error of measurements. The reliability coefficient (R) was 0.962 for weight and 0.973 for height. Nutritional indicators of the study population are presented in Table 1. The nutritional status of most children was within healthy parameters but a few cases of stunting (n = 18, 5.6%), thinness (n = 7, 2.2%) and underweight (n  = 3, 1.3%) were observed. Of the children who were stunted, thin or underweight, girls accounted for 50%, 43% and 67% of the cases, respectively.

No child had a total protein value below the normal range and of 315 children examined, 7 (57% girls) were anemic.

Overall, of 320 children, 33 (10.3%) had at least a form of nutritional deficit. Five of these children (15.2%) were negative for any STH, while 28 (84.8%) were infected with one or more STH. Among the latter, 15 children were monoparasitized, while 13 were polyparasitized.

### Associations between STH infections and nutritional status

Results of the one-way ANOVA analysis revealed that mean values for WAZ scores were significantly lower in children with moderate-to-heavy infections by either *T. trichiura* (*p = *0.020) or *A. lumbricoides* (*p = *0.015) compared to children with no or light infections. This was not observed in the case of hookworm infections, likely due to the fact that the vast majority (94%) of such infections were light. On the other hand, the scores for the other two indicators (HAZ and BAZ) did not differ significantly across the various infection intensities of any of the helminth species.

**Figure 1:**
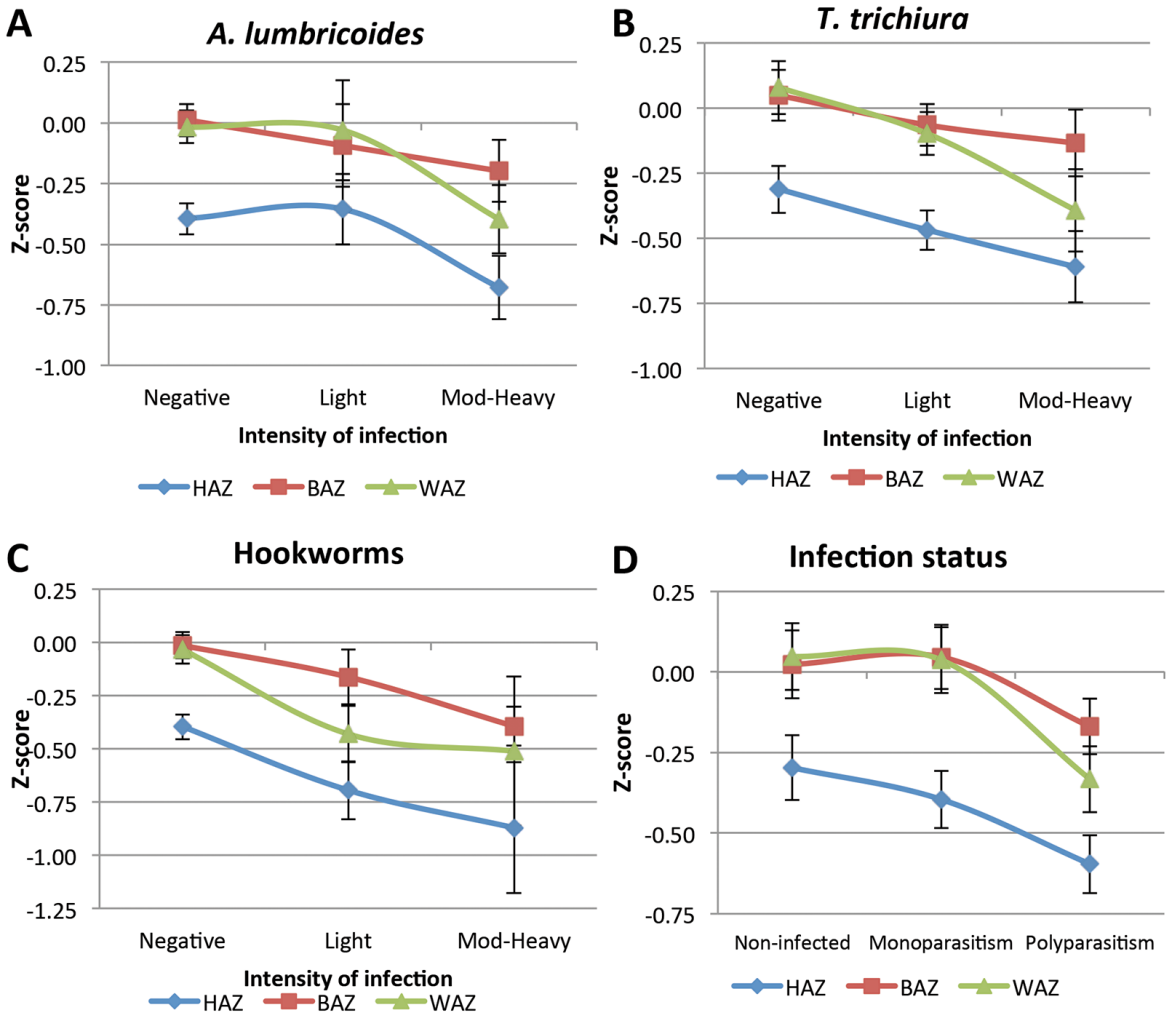
Plots of Z-scores for anthropometric indicators vs. STH intensity of infection/infection status. A decreasing trend in Z-scores was observed as intensity of infection increased (plots A–C) and as the number of species parasitizing increased (plot D). **HAZ:** height-for-age Z-score; **BAZ:** BMI-for-age Z-score; **WAZ:** weight-for-age Z-score. **Mod-Heavy:** infections of moderate-to-heavy intensity.

However, as depicted in Figure 1, a negative trend –although not always significant- between infection intensity and the mean values of all anthropometric indicators was identified. In other words, the heavier the intensity, the lower the HAZ, BAZ and WAZ mean values (Figure 1, plots A, B and C). A similar trend was observed in terms of infection status: as polyparasitism increased, the mean values of all anthropometric indicators decreased (Figure 1, plot D). As data in Table 1 show, this trend was significant in terms of WAZ scores (*p* = 0.012), marginally significant for HAZ scores (*p* = 0.071) but not significant for BAZ scores (*p* = 0.202).

Estimated coefficients (β) from multivariable GEE linear models are shown in Table 3. Compared to no or light infections, moderate-to-heavy infections with any STH were significantly correlated with a decrease in WAZ scores (β = −0.34, 95% CI = −0.62 to −0.06, *p* = 0.018). This correlation was only marginally significant for HAZ scores (β = −0.20, 95% CI = −0.44 to 0.04, *p* = 0.108) but not significant for BAZ (*p* = 0.622). Polyparasitism was found inversely correlated with both WAZ and HAZ scores. For WAZ, this correlation was significant (β = −0.37, 95% CI = −0.66 to −0.08, *p* = 0.012) whereas for HAZ, it was only marginally significant (β = −0.24, 95% CI = −0.50 to 0.02, *p* = 0.074). However, no evidence for association between polyparasitism and BAZ scores was found (*p* = 0.446). With respect to age, there was a strong negative correlation between age and HAZ and BAZ scores (β = −0.16, 95% CI = −0.24 to −0.09, *p*<0.001 and β = −0.12, 95% CI = −0.21 to −0.03, *p* = 0.008, respectively). Conversely, WAZ scores were not correlated with age (*p* = 0.428).

**Table 3:** Adjusted coefficients (β) from multivariable GEE linear models of anthropometric indicators, accounting for within-school clustering.

		HAZ (n = 320)		BAZ (n = 320)		WAZ† (n = 234)	
Variable	Category	Coefficient (β) (95% CI)	*p*-value	Coefficient (β) (95% CI)	*p*-value	Coefficient (β) (95% CI)	*p*-value
Model 1: Infection intensity							
*Age* a	---	−0.16 (−0.24, −0.09)	<0.001	−0.12 (−0.21, −0.03)	0.008	−0.05 (−0.18, 0.08)	0.428
*Sex*	Girls	1		1		1	
	Boys	0.05 (−0.15, 0.26)	0.622	−0.03 (−0.24, 0.19)	0.800	0.16 (−0.07, 0.40)	0.169
*SES* b	---	0.03 (−0.08, 0.13)	0.605	0.08 (−0.03, 0.19)	0.130	0.06 (−0.05, 0.17)	0.296
*Anemia*	Absence	1		1		1	
	Presence	0.08 (−0.80, 0.95)	0.865	0.25 (−0.37, 0.88)	0.428	−0.19 (−0.63, 0.25)	0.399
*Infection intensity*	Negative-to-light	1		1		1	
	Moderate-to-heavy	−0.20 (−0.44, 0.04)	0.108	−0.06 (−0.31, 0.19)	0.622	−0.34 (−0.62, −0.06)	0.018
Model 2: Infection status							
*Age* a	---	−0.16 (−0.24, −0.09)	< 0.001	−0.12 (−0.21, −0.35)	0.006	−0.06 (−0.19, 0.06)	0.326
*Sex*	Girls	1		1		1	
	Boys	0.06 (−0.15, 0.26)	0.568	−0.02 (−0.23, 0.20)	0.872	0.17 (−0.07, 0.40)	0.160
*SES* b	---	0.02 (−0.08, 0.13)	0.659	0.08 (−0.03, 0.18)	0.164	0.05 (−0.06, 0.17)	0.346
*Anemia*	Absence	1		1		1	
	Presence	0.09 (−0.77, 0.95)	0.842	0.23 (−0.37, 0.83)	0.452	−0.17 (−0.53, 0.17)	0.321
*Infection status*	Non-infected	1		1		1	
	Monoparasitism	−0.03 (−0.29, 0.22)	0.793	0.08 (−0.19, 0.36)	0.553	0.02 (−0.27, 0.30)	0.906
	Polyparasitism	−0.24 (−0.50, 0.02)	0.074	−0.10 (−0.37–0.16)	0.446	−0.37 (−0.66, −0.08)	0.012

GEE: Generalized estimating equations.

HAZ: height-for-age Z-score; BAZ: BMI-for-age Z-score; WAZ: weight-for-age Z-score.

† WAZ calculation is not recommended for children >10 years of age.

a Age as continuous variable in years.

b SES: Socio-economic status. Factor constructed as described in ‘Methods’ section.

Multivariable GEE logistic models revealed that age of the studied population was significantly associated with stunting. Children >10 years old were three times more likely to be stunted (OR = 3.31; 95% CI = 1.23–8.90, *p = *0.018) than younger children. Age, on the other hand, was only marginally significantly associated with thinness (*p<*0.15).

Neither infection intensity nor infection status (polyparasitism) was found associated with stunting or thinness. Finally, since only three children were underweight (WAZ <−2SD) no statistical model was produced for this nutritional indicator.

## Discussion

In a little more than a decade, moderate economic progress alongside dedicated efforts for STH control –mainly through national deworming campaigns- have contributed to the decrease of Honduras' national STH prevalence [1]. Yet, as the data from our study show, some rural communities have persistently high STH transmission and perhaps they face greater struggles in overcoming poverty and inequities [17]. Indeed, considering that five of the seven participating schools reported some form of mass-deworming during the past year, a prevalence of 72.5% for any STH among these children is remarkably high. According to the work of Hall and colleagues, a prevalence of 70% or greater carries a high probability of disease [12]. Moreover, 36% of infected children were harbouring moderate-to-heavy infections. The new vision for a world free of childhood morbidity due to these helminths, according to the WHO, is reducing the prevalence of STH infection of moderate and heavy intensity to ≤1% [1]. Therefore, these data alone underscore the need for Honduras to continue and sustain its deworming program, and more importantly, to implement and monitor integrated control efforts [28], [29].

The predominance of *T. trichiura* over *A. lumbricoides* (66.9% versus 30.3%) may indicate that the single-dose albendazole schedule currently used for deworming has been less effective for reducing trichuriasis as this parasite is less susceptible to this drug [30], [31]. Even though it might not be feasible to implement a different PC regimen in Honduras at the moment, it is important to be vigilant of the different patterns of transmission of individual STH species. At the same time, it would be useful to conduct baseline studies to measure reinfection rates [32] and drug efficacy [33], as well as to make efforts to detect potential emergence of resistance to benzimidazoles [34], [35], [36].

In terms of prevalence, our study shows that children older than 10 years of age were more likely to be infected with any STH than younger children, underscoring the importance of deworming children throughout their primary school years [1]. The fact that in our study boys were more likely than girls to harbour hookworm infection warrants further investigation as there might be gender-related factors playing an important role in exposure to hookworms, as previously suggested [37], [38]. Along with high prevalence, we also found a high proportion of polyparasitism with almost half of those infected (44%) harbouring 2 or 3 helminths. This finding is consistent with the epidemiological profile of endemic countries [16], [39], [40], [41] and although already observed in Honduras [6], [42], [43], it has not received sufficient attention in the country. The impact of infections by multiple parasite species has been subject to some attention in the last decade [44] and studies show that concurrent infections may have additive or synergistic detrimental effects, especially in childhood [11], [45]. Given that regular PC interventions will eventually result in reduced infection intensity, light polyparasitic infections will become more relevant [45]. Therefore, addressing polyparasitism in future WHO recommendations merits consideration.

In terms of nutritional status, the majority of studied children were within the WHO reference values for growth and nutrition. This is uncommon for a Honduran rural population [46]. In fact, the proportion of children suffering chronic undernutrition (as measured by stunting) identified in this study was 5.6% well below current national urban (13.7%) or rural (32%) figures. On the other hand, the proportion of global undernutrition (as measured by thinness) was 2.2%, below the national urban (6.2%) or rural (14.8%) averages [47] and very close to the expected value (in a healthy population, approximately 2.1% of individuals will fall either above or below 2SD of the HAZ, BAZ and WAZ reference values). Although assessing food-security was beyond the scope of this study, a possible explanation for this finding is that Catacamas valley and surrounding areas are situated in fertile lands and food insecurity is not as dramatic as in other parts of the country [19], [48].

We found that the risk of stunting increased with age, a phenomenon also found in similar studies conducted in Peru in both school-age children [16] and in pre-schoolers [49], as well as in Malaysia [50], Colombia [51] and Guatemala [39]. It appears, therefore, that once stunted, children continue to be so, even when more acute indicators such as WAZ and BAZ fall within healthy parameters. Longitudinal studies could help elucidate the most favourable moment for children to prevent growth faltering that may lead to stunting.

As mentioned, we aimed to ascertain potential associations of STH infections with the children's nutritional status (stunting, underweight and thinness) but our data did not support such associations. It is recognized that studying the impact of intestinal helminths on child growth and nutrition in endemic populations is not an easy endeavour as it is difficult to control for other environmental or socio-economic factors or seasonal changes in the food supply [4], [12]. It is worth mentioning, however, that SES status (as we measured it) was not found associated with either increased odds of stunting or thinness or with a decrease in anthropometric indicators Z-scores.

Notwithstanding this lack of association in our study, when looking at the distribution of the actual Z-scores for these indicators in the multivariable analyses, we found that both moderate-to-heavy infections with any STH and polyparasitism were significantly associated with lower WAZ scores. Additionally, at the species level, negative trends (albeit not all significant) were observed between STH infection and WAZ, BAZ and HAZ mean scores; namely, as intensity of infection or the number of species parasitizing increased, Z-score mean values for the three measured anthropometric indicators decreased. A similar finding was reported by Ordoñez and Angulo (2002) in a cross-sectional study in which polyparasitized children had lower HAZ and WAZ scores [51]. Thus, examining anthropometric Z-scores values might be useful in providing additional insight into the impact of STH on children's nutritional status as they may reveal subtle changes missed when focusing only on end outcomes (*i.e.*, stunting, thinness and underweight). By the same token, Z-score values may be able to pin-point improvements in children's nutrition after anthelminthic treatment even if significant changes in end outcomes cannot be demonstrated.

Limitations of this study arise from its cross-sectional nature as direct causal relationship between STH and nutritional status cannot be determined. This is why large-scale prospective studies with rigorous design and the corresponding funding are necessary to investigate neglected tropical diseases including helminthiases. Another potential limitation stems from the fact that our investigation was part of a parent study with a sample size calculation based on being able to detect an important difference in sex-specific STH prevalences instead of nutritional indicators and this may limit the precision of our results. We trust that our findings will shed light into the design of future studies in Honduras.

In terms of our parasitological findings, the analysis of a single stool sample may have underestimated STH prevalence in our study but by the high prevalence obtained, this underestimation might be minimal. Further, recent work suggests that Kato-Katz is reasonably accurate for *A. lumbricoides* and *T. trichiura* although less so for hookworms [52]. Likewise, intensity of infection may have been underestimated although recent publications suggest that Kato-Katz results are fairly reliable for the three STH investigated in the present study [52], [53]. Malaria, other intestinal parasites or gastrointestinal infections were not determined and a role for these on children's nutritional status cannot be ruled out. Finally, an important limitation in identifying undernutrition factors is that this study did not entail an exhaustive investigation of underlying causes of malnutrition (*e.g.*, social determinants, food security, dietary intake and expenditure, etc). Future research should address this gap although cross-sectional studies might not be able to reveal concrete answers, as shown by Gray et al. (2006) [46].

Strengths of this study are: including a design effect in the sample size estimation to take into account clustering by school, obtaining a high participation rate, and that our sample is likely representative of the communities' school children as in Honduras 95% children attend primary school [47]. Also, by utilizing laboratory protocols and anthropometric measurements recommended by the WHO, our results permit comparisons with other studies both nationally and internationally.

In conclusion, the prevalence data obtained in this study contribute with accurate and updated information to map out the situation of STH infections in Honduras. Further, this study provides a unique insight into the nutritional status of a cohort of school children living in rural Honduras and, very importantly, it is to our knowledge, the first in the country to explore the potential impact of STH infections on children's nutritional status. Our results also underscore the need for more research into the health effects of polyparasitism in children. It is hoped that the findings presented here will be useful in informing current STH control efforts in Honduras and encourage future investigations that take into account the social and geographical differences across the country.

## Supporting Information

Checklist S1 STROBE Checklist.
(DOC)
