# Supplementary material for: Soil-Transmitted Helminth Infections and Nutritional Status in School-age Children from Rural Communities in Honduras
Source: PLoS Negl Trop Dis. 2013 Aug 8;7(8):e2378. doi: 10.1371/journal.pntd.0002378 (PMC3738480; doi:10.1371/journal.pntd.0002378)
Supplement: Checklist S1 — STROBE Checklist. (DOC) [file pntd.0002378.s001.doc]

|  | **STROBE Checklist (For cross-sectional studies)** | | | |
| --- | --- | --- | --- | --- |
| **Section in manuscript** | | **Item** | **Recommendation** | **Compliance** |
| **TITLE & ABSTRACT** | |  |  |  |
|  | | 1 | (*a*) Indicate the study’s design with a commonly used term in the title or the abstract | ✓ |
|  | |  | (*b*) Provide in the abstract an informative and balanced summary of what was done and what was found | ✓ |
| **INTRODUCTION** | |  |  |  |
| Background / rationale | | 2 | Explain the scientific background and rationale for the investigation being reported | ✓ |
| Objectives | | 3 | State specific objectives, including any prespecified hypotheses | ✓ |
| **METHODS** | |  |  |  |
| Study design | | 4 | Present key elements of study design early in the paper | ✓ |
| Setting | | 5 | Describe the setting, locations, and relevant dates, including periods of recruitment, exposure, follow-up, and data collection | ✓ |
| Participants | | 6 | (a) Give the eligibility criteria, and the sources and methods of selection of participants | ✓ |
| Variables | | 7 | Clearly define all outcomes, exposures, predictors, potential confounders, and effect modifiers. Give diagnostic criteria, if applicable | ✓ |
| Data sources / measurement | | 8* | For each variable of interest, give sources of data and details of methods of assessment (measurement). Describe comparability of assessment methods if there is more than one group | ✓ |
| Bias | | 9 | Describe any efforts to address potential sources of bias | ✓ |
| Study size | | 10 | Explain how the study size was arrived at | ✓ |
| Quantitative variables | | 11 | Explain how quantitative variables were handled in the analyses. If applicable, describe which groupings were chosen and why | ✓ |
| Statistical methods | | 12 | (a) Describe all statistical methods, including those used to control for confounding | ✓ |
|  | |  | (b) Describe any methods used to examine subgroups and interactions | ✓ |
|  | |  | (c) Explain how missing data were addressed | ✓ |
|  | |  | (d) If applicable, describe analytical methods taking account of sampling strategy | ✓ |
|  | |  | (e) Describe any sensitivity analyses | N/A |
| **RESULTS** | |  |  |  |
| Participants | | 13* | (a) Report numbers of individuals at each stage of study—eg numbers potentially eligible, examined for eligibility, confirmed eligible, included in the study, completing follow-up, and analysed | ✓ |
|  | |  | (b) Give reasons for non-participation at each stage | ✓ |
|  | |  | (c) Consider use of a flow diagram | data left in-text |
| Descriptive data | | 14* | (a) Give characteristics of study participants (eg demographic, clinical, social) and information on exposures and potential confounders | ✓ |
|  | |  | (b) Indicate number of participants with missing data for each variable of interest | ✓ |
| Outcome data | | 15* | Report numbers of outcome events or summary measures | ✓ |
| Main results | | 16 | (a) Give unadjusted estimates and, if applicable, confounder-adjusted estimates and their precision (eg, 95% confidence interval). Make clear which confounders were adjusted for and why they were included | ✓ |
|  | |  | (b) Report category boundaries when continuous variables were categorized | ✓ |
|  | |  | (c) If relevant, consider translating estimates of relative risk into absolute risk for a meaningful time period | N/A |
| Other analyses | | 17 | Report other analyses done—eg analyses of subgroups and interactions, and sensitivity analyses | N/A |
| **DISCUSSION** | |  |  |  |
| Key results | | 18 | Summarise key results with reference to study objectives | ✓ |
| Limitations | | 19 | Discuss limitations of the study, taking into account sources of potential bias or imprecision. Discuss both direction and magnitude of any potential bias | ✓ |
| Interpretation | | 20 | Give a cautious overall interpretation of results considering objectives, limitations, multiplicity of analyses, results from similar studies, and other relevant evidence | ✓ |
| Generalisability | | 21 | Discuss the generalisability (external validity) of the study results | ✓ |
| **OTHER INFORMATION** | |  |  |  |
| Funding | | 22 | Give the source of funding and the role of the funders for the present study and, if applicable, for the original study on which the present article is based | ✓ |
